# Sex differences in umbilical artery Doppler indices: a longitudinal study

**DOI:** 10.1186/s13293-018-0174-x

**Published:** 2018-04-18

**Authors:** Christian Widnes, Kari Flo, Tom Wilsgaard, Torvid Kiserud, Ganesh Acharya

**Affiliations:** 1 Women’s Health and Perinatology Research Group, Department of Clinical Medicine, Faculty of Health Sciences, UiT-The Arctic University of Norway, Tromso, Norway; 2 Department of Obstetrics and Gynecology, University Hospital of North Norway, Sykehusveien 38, PO Box 24, N–9038 Tromso, Norway; 3 Department of Community Medicine, Faculty of Health Sciences, UiT-The Arctic University of Norway, Tromso, Norway; 4 Department of Clinical Science, University of Bergen, Bergen, Norway; 5 Department of Obstetrics and Gynecology, Haukeland University Hospital, Bergen, Norway; 6 Department of Clinical Science, Intervention and Technology, Karolinska Institute, Stockholm, Sweden

**Keywords:** Fetal Doppler, Obstetric ultrasound, Placental blood flow, Sex differences, Umbilical artery, Reference ranges

## Abstract

**Background:**

Sexual dimorphism in placental size and function has been described. Whether this influences the clinically important umbilical artery (UA) waveform remains controversial, although a few cross-sectional studies have shown sex differences in UA pulsatility index (PI). Therefore, we tested whether fetal sex influences the UA Doppler indices during the entire second half of pregnancy and aimed to establish sex-specific reference ranges for UA Doppler indices if needed.

**Methods:**

Our main objective was to investigate gestational age-associated changes in UA Doppler indices during the second half of pregnancy and compare the values between male and female fetuses. This was a prospective longitudinal study in women with singleton low-risk pregnancies during 19–40 weeks of gestation. UA Doppler indices were serially obtained at a 4-weekly interval from a free loop of the umbilical cord using color-directed pulsed-wave Doppler ultrasonography. Sex-specific reference intervals were calculated for the fetal heart rate (HR), UA PI, resistance index (RI), and systolic/diastolic ratio (S/D) using multilevel modeling.

**Results:**

Complete data from 294 pregnancies (a total of 1261 observations from 152 male and 142 female fetuses) were available for statistical analysis, and sex-specific reference ranges for the UA Doppler indices and fetal HR were established for the last half of pregnancy. UA Doppler indices were significantly associated with gestational age (*P* < 0.0001) and fetal HR (*P* < 0.0001). Female fetuses had 2–8% higher values for UA Doppler indices than male fetuses during gestational weeks 20^+0^–36^+6^ (*P* < 0.05), but not later. Female fetuses had higher HR from gestational week 26^+0^ until term (*P* < 0.05).

**Conclusions:**

We have determined gestational age-dependent sex differences in UA Doppler indices and fetal HR during the second half of pregnancy, and correspondingly established new sex-specific reference ranges intended for refining diagnostics and monitoring individual pregnancies.

## Background

The importance of conducting longitudinal studies and analyzing data accounting for biological differences related to sex has been highlighted a decade ago [1]. Previous studies have shown sex-specific differences in fetal development regarding growth and adaption to intrauterine environment [2]. Male sex is an independent risk factor for unfavorable perinatal outcomes including fetal distress during labor [3, 4], premature birth [5, 6], adverse neonatal outcome [7], and early neonatal death [2]. This has been referred to as “the male disadvantage” [8] and the female neonatal survival advantage is well recognized [9]. However, the total mortality from conception to birth is greater among female fetuses [10]. The human placenta demonstrates sex-related differences at both structural and functional levels [11, 12]. Both birth weight and placental weight [13] are higher for males compared with females. Sexual dimorphism in the regulation and expression of genes, and signaling pathways [14–17], generate differences in placental function and intrauterine environment that may lead to sex differences in health status later in life [12, 18].

Antenatal growth charts show significant differences in biometrics between male and female fetuses [19]. When using two-dimensional ultrasonography to assess fetal growth, these sex-specific growth charts perform better in identifying small for gestational age (SGA) fetuses [20] at increased risk of fetal demise [21].

Umbilical artery (UA) Doppler indices, i.e., pulsatility index (PI), resistance index (RI), and systolic/diastolic ratio (S/D) calculated from blood flow velocities, are used as an important clinical tool for evaluating fetal wellbeing in high-risk pregnancies and to predict outcome of growth restricted fetuses [22]. Their use in high-risk pregnancies has the potential to reduce obstetric interventions and perinatal deaths [23]. The increased UA PI is a marker of raised placental vascular impedance associated with microvascular lesions [24] and correspondingly reduced placental function. Longitudinal reference ranges for UA Doppler indices calculated from both cross-sectional and serial measurements have previously been published [25, 26]. However, these studies do not take into account possible sex differences.

Doppler ultrasonographic studies exploring fetal sex differences in the ductus venosus during the first trimester have shown antagonistic results [27–29]. Another study performed just prior to active labor, in term pregnancies, demonstrated no differences in UA PI, but statistically significant lower values for the middle cerebral artery (MCA) PI, MCA peak systolic velocity (PSV) and normalized umbilical venous blood flow (Q_uv)_ in male compared with female fetuses [30], but these differences did not translate into differences in perinatal outcome. One recent study of the feto-placental circulation and cardiac function during 28–34 gestational weeks showed higher preload and lower afterload (significantly lower UA PI) in male fetuses [31]. In a cross-sectional study investigating maternal hemodynamics and placental circulation, we recently demonstrated significantly lower UA PI in male compared with that in female fetuses at 22^+0^–24^+0^ weeks of gestation [32].

Based on such observations, the main objective of our present study was to assess the effect of fetal sex on UA Doppler indices during the entire second half of pregnancy and correspondingly establish sex-specific longitudinal reference ranges for clinical use.

## Methods

In this study, we used data from a total of 306 healthy pregnant women with uncomplicated singleton pregnancy participating in three prospective longitudinal observational studies that included investigation of feto-placental hemodynamics. The women, all > 18 years old, were recruited at the time of routine ultrasound screening at 17–20 weeks of gestation at the University Hospital of North Norway. The gestational age was based on the biometry of fetal head performed during this scan. Women with singleton pregnancy with no history of any systemic diseases that may affect the course and outcome of pregnancy were included. A history of preeclampsia, intrauterine growth retardation (IUGR), gestational diabetes or preterm labor before 34 weeks in previous pregnancy, multiple pregnancy, maternal smoking, or presence of any chromosomal or major structural fetal anomaly in the current pregnancy were reasons for exclusion. The study protocols were approved by the Regional Committee for Medical and Health Research Ethics –North Norway (REK Nord 74/2001, 52/2005, and 105/2008), and informed written consent was obtained from each participant.

For Doppler ultrasonography, an Acuson Sequoia 512 ultrasound system with a 6-MHz curvilinear transducer (Mountain View, CA, USA) or a Vivid 7 Dimension ultrasound system equipped with a 4MS sector transducer with frequencies of 1.5–4.3 MHz (GE Vingmed Ultrasound AS, Horten, Norway) was used. Four experienced clinicians performed the examinations at approximately 4-weekly intervals. In two of the studies all measurements were performed by two single operators, and in the third study three different operators did the examinations. The sex of the fetus was neither acknowledged nor recorded prenatally during ultrasonography. Each participant was examined 3–6 times by the same clinician, starting from 19 to 22 gestational weeks until delivery. The estimated fetal weight (EFW) was computed at each visit from measurements of the biparietal diameter (BPD), abdominal circumference (AC), and femur length (FL) based on the Hadlock 2 formula [33]. Blood flow velocity waveforms of the UA were obtained from the free-floating loop of the umbilical cord using pulsed-wave Doppler optimizing the insonation with simultaneous use of color Doppler. The angle of insonation was always kept < 15 degrees, and angle correction was used if the angle was not zero. To ensure Doppler recording of the spatial maximum blood velocity, an expanded sample gate of 5–12 mm was used depending on gestational age. The high-pass filter was set at low. The blood flow velocities (i.e., PSV, end-diastolic velocity (EDV), and time-averaged maximum velocity (TAMXV)), and fetal heart rate (HR) were measured online using the maximum velocity envelope recorded over the cardiac cycle. An average of three consecutive cycles were used for analysis. The ALARA (as low as reasonably achievable) principle [34] was employed. At all times during the ultrasonographic examination the mechanical and thermal indices were kept below 1.9 and 1.5, respectively. We recorded the UA blood flow successfully in 1243 out of 1261 (98.57%) observations. The UA Doppler indices were calculated from the recorded velocities as follows: PI = (PSV - EDV)/TAMXV [35], RI = (PSV - EDV)/PSV [36], and S/D ratio = PSV/EDV [37].

The reproducibility of the Doppler parameters studied, expressed by the intra-observer coefficient of variation (CV) and the inter-observer CV, has previously been assessed and reported [26, 38, 39].

All women had a regular antenatal follow-up according to local guidelines. Following delivery, the course and outcome of pregnancy, including any maternal or fetal complications, gestation at delivery, mode of delivery, birth weight, placental weight, neonatal sex, Apgar scores, umbilical cord blood acid-base status, and neonatal outcome was obtained from the electronic medical records. On the second day post-partum, a pediatrician routinely examined all neonates prior to discharge.

Statistical Analysis Software version 9.3 (SAS institute Inc., Cary, NC, USA) was used for statistical analysis. Logarithmic or power transformations were performed on all numerical variables that were not normally distributed, in order to best meet the criteria of normal distribution. The best transformation for each variable was determined using the Box-Cox regression. Fractional polynominals were used to achieve best-fitting curves in relation to gestational age for each variable, accommodating for nonlinear associations. We used multilevel modeling to construct gestational age-specific reference percentiles from each fitted model according to Royston and Altman [40]. The comparison between groups was done using independent samples *t* test for continuous variables, while the chi-square test was used for categorical variables. The comparison of UA Doppler indices between male and female fetuses was performed for each gestational week after having checked and adjusted for possible confounding factors (fetal HR, EFW, and placental weight) by including a cross-product term between sex and gestational age in the aforementioned multilevel models. The level of statistical significance was set at a two-tailed *p* value of < 0.05.

## Results

From a total study population of 306 women, 12 were excluded because they had < 3 observations, leaving complete data from 294 pregnancies available for statistical analysis. There were 152 male and 142 female fetuses. The baseline characteristics of the study population, including pregnancy and neonatal outcomes, are presented in Table 1. We observed no statistically significant differences between the two groups in any of the listed baseline variables.

**Table 1:** Baseline characteristics of the study population and pregnancy outcomes

	Female (*n* = 142)	Male (*n* = 152)	*P* value
Maternal			
Age (years)	29 (range 20–43)	30 (range 18–40)	0.646
Body mass index at booking (kg/m^2^)	24.85 ± 4.00	24.45 ± 3.65	0.374
Nulliparous	70 (49.3%)	76 (50.0%)	0.904
Fetal			
Gestational age at birth (days)^a^	279 (range 238–297)	280 (range 234–297)	0.633
Birth weight (g)	3593 ± 431	3603 ± 533	0.860
Placental weight (g)	631 ± 128	645 ± 142	0.385
Fetal-placental ratio	5.84 ± 1.03	5.74 ± 0.98	0.413
5-min Apgar score	10 (range 2–10)	9 (range 0–10)	0.348
Umbilical artery pH	7.25 ± 0.10	7.25 ± 0.08	0.831
Umbilical artery base excess (mmol/L)	−4.16 ± 3.91	−4.54 ± 3.06	0.472
Meconium stained liquor	29 (20.4%)	25 (16.6%)	0.443
Admission to NICU	8 (5.7%)	11 (7.3%)	0.577
Preterm birth, < 37^+0^ weeks’ gestation	1 (0.7%)	6 (3.9%)	0.068
Preeclampsia	3 (2.1%)	6 (3.9%)	0.361
SGA/IUGR	1 (0.7%)	3 (2.0%)	0.348
Mode of delivery			
Normal	114 (80.3%)	126 (82.9%)	0.563
Vacuum/forceps	6 (4.2%)	7 (4.6%)	0.874
Cesarean section	22 (15.5%)	19 (12.5%)	0.459

Data are presented as *n* (%), median (range), or mean ± SD, as appropriate

*P* values were calculated using independent samples *t* test for continuous variables and chi-square tests for categorical variables

*NICU* neonatal intensive care unit, *SGA* small for gestational age, *IUGR* intrauterine growth retardation

^a^279 days = 39^+6^ weeks, 280 days = 40^+0^ weeks

There were 650 and 611 observations for male and female fetuses, respectively. The UA Doppler indices (PI, RI, and S/D ratio) and the fetal HR were significantly associated with gestational age (*P* < 0.0001), and there was also an association between UA Doppler indices and fetal HR (*P* < 0.0001). We found no sex differences in EFW at any gestational week, and there was no confounding effect of EFW on UA Doppler indices.

**Fig. 1:**
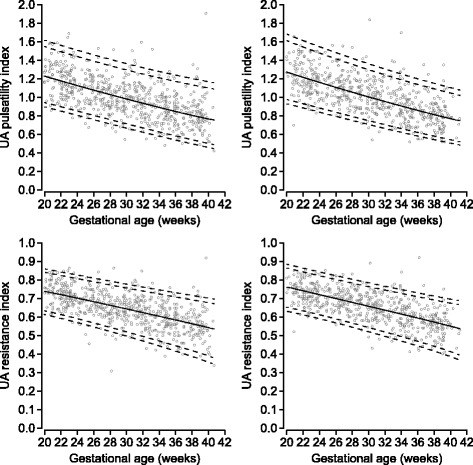
Umbilical artery pulsatility index and resistance index. Sex-specific reference ranges for umbilical artery (UA) pulsatility index and resistance index (left male, right female). The solid line represents the mean, and the interrupted lines represent 2.5th, 5th, 95th, and 97.5th percentiles

**Fig. 2:**
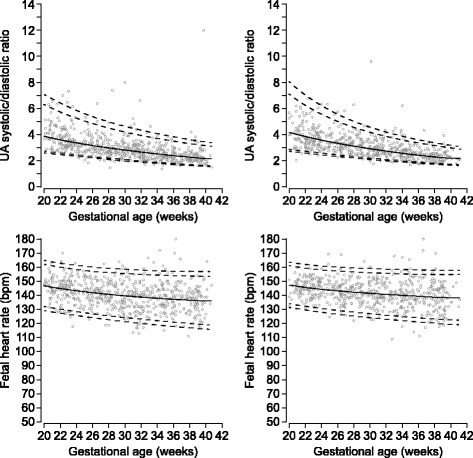
Umbilical artery systolic/diastolic ratio and heart rate. Sex-specific reference ranges for umbilical artery (UA) systolic/diastolic ratio and fetal heart rate (left male, right female). The solid line represents the mean, and the interrupted lines represent 2.5th, 5th, 95th, and 97.5th percentiles

**Fig. 3:**
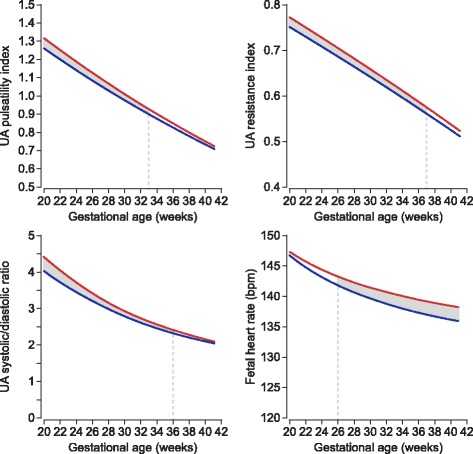
Sex differences in fetal heart rate and umbilical artery Doppler indices adjusted for fetal heart rate. Gestational age-related sex differences in the mean values for umbilical artery (UA) pulsatility index (top left), resistance index (top right), systolic/diastolic ratio (bottom left), all adjusted for fetal heart rate, and fetal heart rate (bottom right) during the second half of pregnancy. The red line represents female, and the blue line represents male. The shaded area indicates significant differences (*P* < 0.05)

Sex-specific reference curves for the UA Doppler indices and the fetal HR for gestational weeks 20–40 are presented in Figs. 1 and 2. The reference values with their respective 2.5th, 5th, 10th, 25th, 50th, 75th, 90th, 95th, and 97.5th percentiles are presented in Tables 2, 3, 4, 5, 6, 7, 8, and 9. The corresponding gestational age-related sex differences in the mean values for UA PI, RI, and S/D ratio, all adjusted for fetal HR, are displayed in Fig. 3, along with the gestational age-specific mean fetal HR for male and female fetuses during the second half of pregnancy.

**Table 2:** Umbilical artery pulsatility index (male)

Gestation (week)	2.5th percentile	5th percentile	10th percentile	25th percentile	Mean	75th percentile	90th percentile	95th percentile	97.5th percentile
20	0.90	0.95	1.01	1.11	1.23	1.35	1.48	1.55	1.62
21	0.88	0.92	0.98	1.08	1.20	1.33	1.45	1.52	1.59
22	0.85	0.90	0.96	1.06	1.18	1.30	1.42	1.49	1.56
23	0.83	0.88	0.93	1.03	1.15	1.27	1.39	1.47	1.53
24	0.81	0.86	0.91	1.01	1.13	1.25	1.37	1.44	1.51
25	0.79	0.83	0.89	0.98	1.10	1.22	1.34	1.42	1.48
26	0.76	0.81	0.86	0.96	1.08	1.20	1.32	1.39	1.46
27	0.74	0.79	0.84	0.94	1.05	1.17	1.29	1.37	1.43
28	0.72	0.76	0.82	0.91	1.03	1.15	1.27	1.34	1.41
29	0.69	0.74	0.79	0.89	1.00	1.13	1.25	1.32	1.39
30	0.67	0.72	0.77	0.87	0.98	1.10	1.22	1.30	1.36
31	0.65	0.70	0.75	0.84	0.96	1.08	1.20	1.28	1.34
32	0.63	0.67	0.73	0.82	0.94	1.06	1.18	1.25	1.32
33	0.61	0.65	0.70	0.80	0.91	1.04	1.16	1.23	1.30
34	0.58	0.63	0.68	0.78	0.89	1.02	1.14	1.21	1.28
35	0.56	0.61	0.66	0.76	0.87	1.00	1.12	1.19	1.26
36	0.54	0.59	0.64	0.73	0.85	0.98	1.10	1.17	1.24
37	0.52	0.57	0.62	0.71	0.83	0.96	1.08	1.16	1.23
38	0.50	0.54	0.60	0.69	0.81	0.94	1.06	1.14	1.21
39	0.48	0.52	0.58	0.67	0.79	0.92	1.04	1.12	1.19
40	0.46	0.50	0.56	0.65	0.77	0.90	1.02	1.10	1.17

Sex-specific reference values of the umbilical artery pulsatility index (UA PI) for the 2.5th, 5th, 10th, 25th, 50th, 75th, 90th, 95th, and 97.5th percentiles during the second half of pregnancy (male fetuses)

**Table 3:** Umbilical artery pulsatility index (female)

Gestation (week)	2.5th percentile	5th percentile	10th percentile	25th percentile	Mean	75th percentile	90th percentile	95th percentile	97.5th percentile
20	0.93	0.98	1.04	1.15	1.27	1.41	1.53	1.61	1.68
21	0.91	0.96	1.02	1.12	1.25	1.38	1.50	1.58	1.65
22	0.89	0.94	0.99	1.10	1.22	1.35	1.47	1.54	1.61
23	0.86	0.91	0.97	1.07	1.19	1.32	1.44	1.51	1.58
24	0.84	0.89	0.94	1.04	1.16	1.29	1.41	1.48	1.55
25	0.82	0.86	0.92	1.02	1.13	1.26	1.38	1.45	1.51
26	0.80	0.84	0.90	0.99	1.11	1.23	1.35	1.42	1.48
27	0.77	0.82	0.87	0.97	1.08	1.20	1.32	1.39	1.45
28	0.75	0.80	0.85	0.94	1.06	1.17	1.29	1.36	1.42
29	0.73	0.77	0.83	0.92	1.03	1.15	1.26	1.33	1.39
30	0.71	0.75	0.80	0.90	1.00	1.12	1.23	1.30	1.37
31	0.69	0.73	0.78	0.87	0.98	1.10	1.21	1.28	1.34
32	0.67	0.71	0.76	0.85	0.96	1.07	1.18	1.25	1.31
33	0.64	0.69	0.74	0.83	0.93	1.04	1.15	1.22	1.28
34	0.62	0.67	0.71	0.80	0.91	1.02	1.13	1.20	1.26
35	0.60	0.64	0.69	0.78	0.88	1.00	1.10	1.17	1.23
36	0.58	0.62	0.67	0.76	0.86	0.97	1.08	1.15	1.21
37	0.56	0.60	0.65	0.74	0.84	0.95	1.05	1.12	1.18
38	0.54	0.58	0.63	0.71	0.82	0.92	1.03	1.10	1.16
39	0.52	0.56	0.61	0.69	0.79	0.90	1.01	1.07	1.13
40	0.51	0.54	0.59	0.67	0.77	0.88	0.98	1.05	1.11

Sex-specific reference values of the umbilical artery pulsatility index (UA PI) for the 2.5th, 5th, 10th, 25th, 50th, 75th, 90th, 95th, and 97.5th percentiles during the second half of pregnancy (female fetuses)

**Table 4:** Umbilical artery resistance index (male)

Gestation (week)	2.5th percentile	5th percentile	10th percentile	25th percentile	Mean	75th percentile	90th percentile	95th percentile	97.5th percentile
20	0.61	0.64	0.66	0.70	0.74	0.78	0.82	0.84	0.86
21	0.60	0.62	0.65	0.69	0.73	0.77	0.81	0.83	0.85
22	0.59	0.61	0.64	0.68	0.72	0.76	0.80	0.82	0.84
23	0.58	0.60	0.63	0.67	0.71	0.76	0.79	0.82	0.84
24	0.57	0.59	0.62	0.66	0.70	0.75	0.78	0.81	0.83
25	0.56	0.58	0.61	0.65	0.69	0.74	0.78	0.80	0.82
26	0.55	0.57	0.60	0.64	0.68	0.73	0.77	0.79	0.81
27	0.53	0.56	0.58	0.63	0.67	0.72	0.76	0.78	0.80
28	0.52	0.55	0.57	0.62	0.66	0.71	0.75	0.77	0.79
29	0.51	0.53	0.56	0.61	0.65	0.70	0.74	0.77	0.79
30	0.50	0.52	0.55	0.60	0.64	0.69	0.73	0.76	0.78
31	0.48	0.51	0.54	0.58	0.63	0.68	0.73	0.75	0.77
32	0.47	0.50	0.53	0.57	0.62	0.67	0.72	0.74	0.76
33	0.46	0.48	0.51	0.56	0.61	0.66	0.71	0.73	0.76
34	0.44	0.47	0.50	0.55	0.60	0.66	0.70	0.73	0.75
35	0.43	0.46	0.49	0.54	0.59	0.65	0.69	0.72	0.74
36	0.42	0.45	0.48	0.53	0.58	0.64	0.68	0.71	0.73
37	0.40	0.43	0.46	0.52	0.57	0.63	0.67	0.70	0.73
38	0.39	0.42	0.45	0.51	0.56	0.62	0.67	0.69	0.72
39	0.37	0.40	0.44	0.49	0.55	0.61	0.66	0.69	0.71
40	0.36	0.39	0.43	0.48	0.54	0.60	0.65	0.68	0.70

Sex-specific reference values of the umbilical artery resistance index (UA RI) for the 2.5th, 5th, 10th, 25th, 50th, 75th, 90th, 95th, and 97.5th percentiles during the second half of pregnancy (male fetuses)

**Table 5:** Umbilical artery resistance index (female)

Gestation (week)	2.5th percentile	5th percentile	10th percentile	25th percentile	Mean	75th percentile	90th percentile	95th percentile	97.5th percentile
20	0.63	0.65	0.68	0.72	0.76	0.80	0.84	0.86	0.88
21	0.62	0.64	0.67	0.71	0.75	0.79	0.83	0.85	0.87
22	0.61	0.63	0.66	0.70	0.74	0.78	0.82	0.84	0.86
23	0.60	0.62	0.65	0.69	0.73	0.77	0.81	0.83	0.85
24	0.59	0.61	0.64	0.68	0.72	0.76	0.80	0.82	0.84
25	0.58	0.60	0.62	0.67	0.71	0.75	0.79	0.81	0.83
26	0.57	0.59	0.61	0.65	0.70	0.74	0.78	0.80	0.82
27	0.56	0.58	0.60	0.64	0.69	0.73	0.77	0.79	0.81
28	0.54	0.57	0.59	0.63	0.68	0.72	0.76	0.78	0.80
29	0.53	0.56	0.58	0.62	0.67	0.71	0.75	0.77	0.79
30	0.52	0.54	0.57	0.61	0.66	0.70	0.74	0.76	0.78
31	0.51	0.53	0.56	0.60	0.65	0.69	0.73	0.75	0.77
32	0.50	0.52	0.55	0.59	0.64	0.68	0.72	0.74	0.76
33	0.48	0.51	0.53	0.58	0.63	0.67	0.71	0.74	0.76
34	0.47	0.49	0.52	0.57	0.61	0.66	0.70	0.73	0.75
35	0.46	0.48	0.51	0.55	0.60	0.65	0.69	0.72	0.74
36	0.44	0.47	0.50	0.54	0.59	0.64	0.68	0.71	0.73
37	0.43	0.45	0.48	0.53	0.58	0.63	0.67	0.70	0.72
38	0.41	0.44	0.47	0.52	0.57	0.62	0.66	0.69	0.71
39	0.40	0.43	0.46	0.51	0.56	0.61	0.65	0.68	0.70
40	0.38	0.41	0.44	0.49	0.55	0.60	0.65	0.67	0.69

Sex-specific reference values of the umbilical artery resistance index (UA RI) for the 2.5th, 5th, 10th, 25th, 50th, 75th, 90th, 95th, and 97.5th percentiles during the second half of pregnancy (female fetuses)

**Table 6:** Umbilical artery systolic/diastolic ratio (male)

Gestation (week)	2.5th percentile	5th percentile	10th percentile	25th percentile	Mean	75th percentile	90th percentile	95th percentile	97.5th percentile
20	2.6	2.7	2.9	3.3	3.9	4.6	5.5	6.3	7.1
21	2.5	2.7	2.8	3.2	3.7	4.4	5.3	6.0	6.7
22	2.4	2.6	2.8	3.1	3.6	4.3	5.1	5.7	6.4
23	2.4	2.5	2.7	3.0	3.5	4.1	4.9	5.5	6.1
24	2.3	2.4	2.6	2.9	3.4	4.0	4.7	5.3	5.8
25	2.2	2.4	2.5	2.8	3.3	3.8	4.5	5.0	5.6
26	2.2	2.3	2.5	2.8	3.2	3.7	4.4	4.9	5.4
27	2.1	2.2	2.4	2.7	3.1	3.6	4.2	4.7	5.2
28	2.1	2.2	2.3	2.6	3.0	3.5	4.1	4.5	5.0
29	2.0	2.1	2.3	2.5	2.9	3.4	3.9	4.3	4.8
30	2.0	2.1	2.2	2.5	2.8	3.3	3.8	4.2	4.6
31	1.9	2.0	2.1	2.4	2.7	3.2	3.7	4.1	4.5
32	1.9	2.0	2.1	2.3	2.7	3.1	3.6	3.9	4.3
33	1.8	1.9	2.0	2.3	2.6	3.0	3.5	3.8	4.2
34	1.8	1.9	2.0	2.2	2.5	2.9	3.4	3.7	4.1
35	1.7	1.8	1.9	2.2	2.5	2.8	3.3	3.6	3.9
36	1.7	1.8	1.9	2.1	2.4	2.8	3.2	3.5	3.8
37	1.7	1.8	1.9	2.1	2.3	2.7	3.1	3.4	3.7
38	1.6	1.7	1.8	2.0	2.3	2.6	3.0	3.3	3.6
39	1.6	1.7	1.8	2.0	2.2	2.6	2.9	3.2	3.5
40	1.6	1.6	1.7	1.9	2.2	2.5	2.9	3.1	3.4

Sex-specific reference values of the umbilical artery systolic/diastolic (UA S/D) ratio for the 2.5th, 5th, 10th, 25th, 50th, 75th, 90th, 95th, and 97.5th percentiles during the second half of pregnancy (male fetuses)

**Table 7:** Umbilical artery systolic/diastolic ratio (female)

Gestation (week)	2.5th percentile	5th percentile	10th percentile	25th percentile	Mean	75th percentile	90th percentile	95th percentile	97.5th percentile
20	2.7	2.9	3.1	3.5	4.2	5.0	6.2	7.1	8.1
21	2.6	2.8	3.0	3.4	4.0	4.8	5.8	6.6	7.5
22	2.6	2.7	2.9	3.3	3.8	4.6	5.5	6.2	7.0
23	2.5	2.6	2.8	3.2	3.7	4.4	5.2	5.9	6.6
24	2.4	2.6	2.7	3.1	3.6	4.2	5.0	5.6	6.2
25	2.4	2.5	2.7	3.0	3.4	4.0	4.7	5.3	5.8
26	2.3	2.4	2.6	2.9	3.3	3.9	4.5	5.0	5.5
27	2.3	2.4	2.5	2.8	3.2	3.7	4.3	4.8	5.3
28	2.2	2.3	2.5	2.7	3.1	3.6	4.2	4.6	5.0
29	2.1	2.3	2.4	2.6	3.0	3.5	4.0	4.4	4.8
30	2.1	2.2	2.3	2.6	2.9	3.3	3.8	4.2	4.6
31	2.0	2.1	2.3	2.5	2.8	3.2	3.7	4.0	4.4
32	2.0	2.1	2.2	2.4	2.7	3.1	3.6	3.9	4.2
33	1.9	2.0	2.2	2.4	2.7	3.0	3.4	3.7	4.0
34	1.9	2.0	2.1	2.3	2.6	2.9	3.3	3.6	3.9
35	1.9	1.9	2.0	2.2	2.5	2.8	3.2	3.5	3.7
36	1.8	1.9	2.0	2.2	2.4	2.8	3.1	3.4	3.6
37	1.8	1.9	2.0	2.1	2.4	2.7	3.0	3.3	3.5
38	1.7	1.8	1.9	2.1	2.3	2.6	2.9	3.2	3.4
39	1.7	1.8	1.9	2.0	2.3	2.5	2.8	3.1	3.3
40	1.7	1.7	1.8	2.0	2.2	2.5	2.8	3.0	3.2

Sex-specific reference values of the umbilical artery systolic/diastolic (UA S/D) ratio for the 2.5th, 5th, 10th, 25th, 50th, 75th, 90th, 95th, and 97.5th percentiles during the second half of pregnancy (female fetuses)

**Table 8:** Fetal heart rate (male), beats per minute

Gestation (week)	2.5th percentile	5th percentile	10th percentile	25th percentile	Mean	75th percentile	90th percentile	95th percentile	97.5th percentile
20	129	132	135	141	147	153	159	162	165
21	128	131	134	140	146	152	157	161	164
22	127	130	133	139	145	151	156	160	163
23	127	129	133	138	144	150	156	159	162
24	126	129	132	137	143	149	155	158	161
25	125	128	131	136	142	149	154	158	160
26	124	127	130	136	142	148	154	157	160
27	123	126	130	135	141	147	153	156	159
28	123	126	129	134	141	147	153	156	159
29	122	125	128	134	140	146	152	156	159
30	121	124	128	133	140	146	152	155	158
31	121	124	127	133	139	146	152	155	158
32	120	123	127	132	139	145	151	155	158
33	120	123	126	132	138	145	151	155	158
34	119	122	125	131	138	145	151	154	158
35	118	121	125	131	138	144	151	154	157
36	118	121	125	131	137	144	150	154	157
37	117	120	124	130	137	144	150	154	157
38	117	120	124	130	137	144	150	154	157
39	116	120	123	129	136	143	150	154	157
40	116	119	123	129	136	143	150	154	157

Sex-specific reference values of the fetal heart rate (HR) for the 2.5th, 5th, 10th, 25th, 50th, 75th, 90th, 95th, and 97.5th percentiles during the second half of pregnancy (male fetuses)

**Table 9:** Fetal heart rate (female), beats per minute

Gestation (week)	2.5th percentile	5th percentile	10th percentile	25th percentile	Mean	75th percentile	90th percentile	95th percentile	97.5th percentile
20	131	134	137	142	147	153	158	161	164
21	131	133	136	141	146	152	157	160	163
22	130	132	135	140	146	151	156	159	162
23	129	132	134	139	145	151	156	159	161
24	128	131	134	139	144	150	155	158	161
25	127	130	133	138	144	150	155	158	161
26	127	129	132	138	143	149	154	157	160
27	126	129	132	137	143	149	154	157	160
28	125	128	131	136	142	148	154	157	160
29	125	127	131	136	142	148	153	156	159
30	124	127	130	135	141	147	153	156	159
31	124	126	130	135	141	147	153	156	159
32	123	126	129	135	141	147	152	156	159
33	123	125	129	134	140	147	152	156	159
34	122	125	128	134	140	146	152	156	159
35	122	124	128	133	140	146	152	155	158
36	121	124	127	133	139	146	152	155	158
37	121	124	127	133	139	146	152	155	158
38	120	123	127	132	139	145	151	155	158
39	120	123	126	132	139	145	151	155	158
40	120	123	126	132	138	145	151	155	158

Sex-specific reference values of the fetal heart rate (HR) for the 2.5th, 5th, 10th, 25th, 50th, 75th, 90th, 95th, and 97.5th percentiles during the second half of pregnancy (female fetuses)

The results for the gestational age-specific sex differences for fetal HR and, for the adjusted UA Doppler indices, are shown in Table 10. We found significant differences in UA PI, RI, S/D ratio, and HR between male and female fetuses. Female fetuses had significantly higher values for UA PI (range 2.1–4.2%), RI (range 1.7–3.3%), and S/D ratio (range 4.0–8.1%) from 20^+0^ weeks to 32^+6^ and 36^+6^ and 35^+6^ weeks, respectively, but equalized towards term (40 weeks of gestation). For fetal HR, the mean values were similar between male and female fetuses from 20^+0^ to 25^+6^ weeks, but a divergent trend was observed thereafter with the female fetuses showing higher HR (range 0.7–2.2%) compared with male fetuses.

**Table 10:** Level of significance for sex differences in umbilical artery Doppler indices and fetal heart rate

Gestation (week)	UA PI^a^	UA RI^a^	UA S/D ratio^a^	Fetal HR
	*P* value	*P* value	*P* value	*P* value
20	0.00795	0.00282	0.00213	0.58551
21	0.00695	0.00219	0.00171	0.44020
22	0.00621	0.00172	0.00139	0.30634
23	0.00573	0.00139	0.00117	0.19662
24	0.00552	0.00117	0.00103	0.11804
25	0.00564	0.00104	0.00097	0.06888
26	0.00616	0.00102	0.00100	0.04136
27	0.00724	0.00109	0.00113	0.02704
28	0.00915	0.00132	0.00141	0.01991
29	0.01234	0.00178	0.00196	0.01656
30	0.01748	0.00264	0.00295	0.01526
31	0.02550	0.00421	0.00473	0.01518
32	0.03755	0.00699	0.00784	0.01586
33	0.05486	0.01172	0.01309	0.01706
34	0.07849	0.01939	0.02150	0.01864
35	0.10911	0.03110	0.03424	0.02049
36	0.14679	0.04786	0.05239	0.02253
37	0.19098	0.07041	0.07670	0.02470
38	0.24064	0.09901	0.10746	0.02696
39	0.29443	0.13342	0.14444	0.02926
40	0.35092	0.17300	0.18692	0.03159
Overall^b^	0.07560	0.01850	0.01980	0.02560

The results for the gestational age-specific sex differences in mean values for fetal heart rate (HR) and for the adjusted umbilical artery (UA) Doppler indices, organized by gestational week

*UA* umbilical artery, *PI* pulsatility index, *RI* resistance index, *S/D ratio* systolic/diastolic ratio, *HR* heart rate

^a^Adjusted for fetal heart rate

^b^Overall level of significance for sex differences during 20–40 weeks of gestation

## Discussion

The present longitudinal study has demonstrated significant sex differences in UA Doppler indices, female fetuses having significantly more pulsatile waveform than male fetuses during gestational weeks 20^+0^–36^+6^ but not thereafter. The magnitude of effect ranged between 2.1 and 4.2% for the UA PI. Correspondingly, the study provided sex-specific reference ranges for 20–40 weeks’ gestation for the most commonly used indices. As for the fetal HR, the pattern was different; male and female fetuses had similar HR from 20^+0^ to 25^+6^ weeks, but thereafter, the female fetuses had significantly higher HR.

The strength of the study is its longitudinal design and a relatively large sample size (650 observations for male and 611 for female fetuses) providing sufficient power to discover significant sex differences and to construct robust sex-specific reference ranges. The prospective longitudinal design with serial measurements at reasonably spaced intervals during pregnancy is preferable to a cross-sectional design for constructing reference intervals since it better reflects the development during gestation and, in our case, improves the precision of individual participants’ observations. The limitations of our study are related to technical issues concerning UA Doppler velocimetry, and the data being collected from three separate studies, with different operators. However, all the measurements were obtained at a free loop of umbilical cord, under fetal quiescence, keeping the angle of insonation as low as possible (always < 15°). The intra-observer CV for UA PI, RI, and S/D ratio were 10.5, 6.8, and 13.0%, respectively [26].

This study confirms the findings of previous cross-sectional studies that report sex differences in UA Doppler indices during the second and third trimester of pregnancy [31, 32] and that these differences tapered off towards term [30]. However, we were not able to establish at what time in gestation these differences emerged. It would have been desirable to have serial measurements starting from early pregnancy.

Our findings add weight to the recognition of sex differences in fetal development and adaption to the intrauterine environment. The male disadvantage in perinatal outcome when it comes to fetal distress during labor [3, 4], premature birth [5, 6], adverse neonatal outcome [7], and early neonatal death [2] is well documented. It is also well documented that there are significant sex differences in growth of estimated fetal weight [19], birth weight, and placental weight [13], and male and female fetuses have significant differences in growth patterns of individual biometric measurements [41]. Such differences in growth dynamics corroborate the findings of Orzack et al. [10] who found that the unbiased male/female ratio at conception had increased at birth due to a higher female mortality during pregnancy. However, male and female mortality during pregnancy had temporal differences causing undulations in the sex ratio. These findings constitute a plethora of details in which our circulatory results add another piece of evidence to sex differentiation being reflected in all organ systems.

With this background, our finding that there was no significant effect of fetal sex on fetal growth (i.e., EFW) is unexpected, as a recent multinational study showed that fetal sex had an effect of 3.5–4.5% on EFW [42]. However, that study had a considerably higher power than our present study. One can therefore speculate that the present finding of no sex effect on fetal weight could be due to chance, or, as shown in the recent WHO study, due to variations in growth patterns. However, the negligible (10 g) difference in birth weight we observed between the sexes (3593 vs. 3603 g) corroborates our intrauterine growth estimates and ensures that the effect on the Doppler indices was due to sex differences. The issue is important because a difference in size could possibly have explained some of the results. It is interesting, however, that a previous study found “no meaningful correlation between fetal weight and impedance indices” [43].

Mechanisms associated with potential male susceptibility are difficult to underpin. Male fetuses appear to prioritize growth to a greater extent than females and continue to grow in spite of unfavorable intrauterine environment [14]. This may put them at higher risk due to lack of reserve. A higher UA PI, as we have observed in females, could result in a reduction in fetal growth velocity and thereby reduce the risk of adverse outcomes. The mechanisms behind the observed differences in UA Doppler indices are not clear. Slightly higher UA Doppler indices cannot be equated to reduced placental function, and these differences were less pronounced close to term. However, it has been shown that male fetuses born at 24–28 weeks of gestation have more peripheral vasodilatation compared to female fetuses [44]. Furthermore, pregnant women carrying male fetuses are reported to have higher angiotensin (Ang) 1–7 to Ang II ratio in the second trimester [45]. As Ang II is a potent vasoconstrictor and Ang 1–7 is a known vasodilator, relative vasodilatation of placental vessels could be responsible for lower UA PI, RI, and S/D ratio observed in male fetuses.

UA Doppler indices, a surrogate for placental impedance [46], have proved valuable in assessing fetal wellbeing and have the potential to save lives [23]. However, these relations are not consistent [47], as shown in sheep experiments [48, 49]. Although PI increases when embolization causes reduction in vascular cross-section, comparable reduction in vascular cross-section due to angiotensin II did not increase the PI and could even decrease the PI while vascular resistance increased. The reason for this may be a difference in vessel geometry that could impact the wave reflection, a major modifier of the arterial waveform [50, 51]. Thus, the exact mechanism behind the sex difference in the UA pulsatility is not certain.

Another significant finding in the present study was the relatively higher HR in female compared with male fetuses after 26^+ 0^ weeks of gestation, a difference that increased with gestational age (Fig. 3). Higher HR among female fetuses has also been reported previously by others [31, 52]. A plausible cause for having different heart rates in male and female fetuses is differences in hormone levels and rate of maturation of their autonomic nervous system. Higher heart rate variability [53], more complex heart rate patterns [54], and higher catecholamine levels observed in female compared to male fetuses could explain these differences.

In fetal sheep experiments, Morrow et al. demonstrated a significant inverse correlation between the UA Doppler indices (PI, RI, and S/D ratio) and HR [51]. When the HR increased, the UA Doppler indices decreased. We found both higher HR and UA PI, RI, and S/D ratio in female fetuses compared to males, but while the sex differences in HR increased as the pregnancy advanced, the sex differences in the Doppler indices decreased and ceased to exist by term. When we adjusted the gestational age-related sex differences in the mean values for UA PI, RI, and S/D ratio for the fetal HR, the effect size actually increased, i.e., the sexual dimorphism in the UA Doppler indices became more prominent.

Several studies have shown a male preponderance when abnormal UA Doppler waveform is used as a marker of placental dysfunction in pregnancies with IUGR [55, 56]. Increased UA PI correlates with reduced feto-placental perfusion [57] and the degree of microvascular lesions in the placenta [24]. Use of sex-specific reference intervals of UA Doppler indices could potentially improve the identification of pregnancies with placental dysfunction.

## Conclusions

We have demonstrated gestational age-dependent sex differences in UA Doppler indices during the second half of physiological pregnancies and therefore established sex-specific reference ranges. Although the sex difference is modest (2–8%), we believe such references are useful for refining prediction and monitoring of risk pregnancies at a time when such parameters easily are added into software applications increasingly used in clinical practice, particularly since individualized diagnostics and management is an issue.

## Abbreviations

AC: Abdominal circumference
ALARA: As low as reasonably achievable
BMI: Body mass index
CV: Coefficient of variation
EDV: End-diastolic velocity
EFW: Estimated fetal weight
FL: Femur length
HR: Heart rate
IUGR: Intrauterine growth retardation
MCA: Middle cerebral artery
PI: Pulsatility index
PSV: Peak systolic velocity
Q_uv_: Umbilical venous blood flow
RI: Resistance index
S/D ratio: Systolic/diastolic ratio
SD: Standard deviation
SGA: Small for gestational age
TAMXV: Time-averaged maximum velocity
UA: Umbilical artery
